# Editorial: Computational methods for analysis of DNA methylation data, volume II

**DOI:** 10.3389/fbinf.2024.1415791

**Published:** 2024-05-03

**Authors:** Pietro Di Lena, Christine Nardini, Matteo Pellegrini

**Affiliations:** ^1^ Department of Computer Science and Engineering, University of Bologna, Bologna, Italy; ^2^ CNR IAC “Mauro Picone”, Consiglio Nazionale delle Ricerche (CNR), Rome, Italy; ^3^ Department of Molecular, Cell and Developmental Biology, University of California, Los Angeles, Los Angeles, CA, United States

**Keywords:** DNA methylation, DNA methylation age, epigenetic clocks, age acceleration, enrichment analysis

DNA methylation stands out as one of the most extensively investigated epigenetic modifications within the realm of eukaryotic biology. DNA methylation-based predictive models for chronological age, referred to as *epigenetic clocks*, serve as widely used tools for investigating age-related pathologies and physiological alterations. Discrepancies between predicted and actual chronological ages are frequently interpreted as manifestations of biological age acceleration, a phenomenon linked to the onset of various disorders. A plethora of epigenetic clocks have been developed in the literature (Di Lena et al., 2021), and several studies have demonstrated associations between epigenetic age acceleration and pathological conditions (Horvath and Raj, 2018). This active area of research is currently engaged in endeavors to enhance the predictive capabilities of epigenetic clocks and facilitate the translation of their applications into the realm of predictive medicine. Following the significant interest garnered by the first volume of this Research Topic, we are pleased to introduce the second volume. This edition encompasses five contributions dedicated to exploring advancements and challenges in the development of DNA methylation-based epigenetic clocks, as well as examining the applications of epigenetic clocks and DNA methylation analysis in studying disease biology.

In the context of epigenetic clock development, Sala et al. delved into the impact of covariates, such as sex and tissue specificities, as well as training parameters, including the size of the training set and the linear regression model utilized, on the performance of epigenetic clocks. The authors showed that the size of the training set significantly influences prediction performance, as expected. Sex specificity does not substantially affect clock performance, as evidenced by the lack of statistically significant differences between sex-specific and sex-generic linear regression clocks. Conversely, tissue-specific clocks demonstrate superior performance compared to multi-tissue clocks, typically trained on a majority of blood samples. Moreover, the widely utilized elastic-net regression model exhibits comparable or superior prediction performance relative to *ridge* and *lasso* penalization models. These findings offer valuable insights for the development of linear regression epigenetic clocks with enhanced performance capabilities.

A complementary analysis of regression models was provided by Farrell et al. who compared the performance of epigenetic clocks based on penalized linear regression models with that of the non-linear epigenetic pacemaker (EPM) model (Farrell et al., 2020). The EPM model considers DNA methylation as a function of a time-dependent epigenetic state. Differently from linear regression models, the epigenetic state is influenced not only by age but also by other factors, such as sex and cell composition. The authors applied both models to a study on polybrominated biphenyl (PBB) exposure to predict epigenetic age dependent on PBB exposure. They found that both models perform well, with the EPM model showing superior performance. Importantly, only the EPM model identifies significant associations with PBB exposure, highlighting its robustness in investigating factors impacting age acceleration that may be obscured by linear regression models.

A novel regression model for epigenetic clocks, BayesAge, was introduced by Mboning et al. BayesAge was tailored for bisulfite sequencing data. BayesAge utilizes maximum-likelihood estimation (MLE) to address missing data issues and can estimate error bounds, enhancing age inference reliability. Furthermore, BayesAge incorporates LOWESS (LOcally WEighted Scatterplot Smoothing) to capture non-linear associations between DNA methylation data and age. Performance comparisons on down-sampled data indicate superior performance of BayesAge over other linear and non-linear regression models, representing a promising advancement in epigenetic age prediction.

It is well established that several diseases, including cancer (Dugué et al., 2017) and infection with human immunodeficiency virus type 1 (HIV-1) (Horvath and Levine, 2015), are associated with accelerated aging. Two papers within this Research Topic scrutinize the effect of Highly Active Anti-Retroviral Therapy (HAART) on DNA methylation and biological aging. Sehl et al. employed different epigenetic clocks to analyze age acceleration in people living with HIV before and after the initiation of HAART. They discovered that epigenetic aging decreases after HAART initiation but remains persistently greater than that of age-matched seronegative controls. The authors further demonstrated that the magnitude of acceleration is associated with cumulative viral load and changes in T-cell subsets. In parallel, Zhang et al. analyzed the epigenomic-wide changes associated with the initiation of HAART in people living with HIV. They identified CpGs, unrelated to HIV viral load, significantly associated with HAART initiation by comparing DNA methylation profiles of people living with HIV shortly before HAART and post-HAART against seronegative controls. Epigenome-wide association study (EWAS) analysis of such CpGs elucidates that HAART initiation alters DNA methylation in genes associated with immune response and HIV infection. Moreover, enrichment analysis detects Gene Ontologies related to transplant rejection, transplant-related diseases, and other immunologic signatures. Collectively, these findings provide insights into potential biological functions associated with DNA methylation changes induced by HAART.

The most significant conclusion drawn from the first and second volumes of this Research Topic is that although DNA methylation analysis shows great potential, there is a pressing need for further investigation and refinement of methodologies to fully harness its predictive power and translate its findings into actionable insights for clinical practice.
